# Paired Stimulation to Promote Lasting Augmentation of Corticospinal Circuits

**DOI:** 10.1155/2016/7043767

**Published:** 2016-10-09

**Authors:** Noam Y. Harel, Jason B. Carmel

**Affiliations:** ^1^James J. Peters Veterans Affairs Medical Center, Bronx, NY, USA; ^2^Icahn School of Medicine at Mount Sinai, New York, NY, USA; ^3^Burke Medical Research Institute, White Plains, NY, USA; ^4^Weill Cornell Medical College, New York, NY, USA

## Abstract

After injury, electrical stimulation of the nervous system can augment plasticity of spared or latent circuits through focal modulation. Pairing stimulation of two parts of a spared circuit can target modulation more specifically to the intended circuit. We discuss 3 kinds of paired stimulation in the context of the corticospinal system, because of its importance in clinical neurorehabilitation. The first uses principles of Hebbian plasticity: by altering the stimulation timing of presynaptic neurons and their postsynaptic targets, synapse function can be modulated up or down. The second form uses synchronized presynaptic inputs onto a common synaptic target. We dub this a “convergent” mechanism, because stimuli have to converge on a common target with coordinated timing. The third form induces focal modulation by tonic excitation of one region (e.g., the spinal cord) during phasic stimulation of another (e.g., motor cortex). Additionally, endogenous neural activity may be paired with exogenous electrical stimulation. This review addresses what is known about paired stimulation of the corticospinal system of both humans and animal models, emphasizes how it qualitatively differs from single-site stimulation, and discusses the gaps in knowledge that must be addressed to maximize its use and efficacy in neurorehabilitation.

## 1. Introduction

Many skills, including those most elemental to survival—eating, fleeing, and attracting mates—must be learned by associating a context with a function. The “rules” of synaptic learning have largely been gleaned from studies of the hippocampus and neocortex, both of which are highly adapted for learning. Three fundamental processes enable associative learning. First, learning depends on the relative firing of an input neuron and a receiving neuron that are connected by a synapse. This type of learning, originally proposed by Hebb, is known as Hebbian or spike-timing dependent plasticity [1, 2]. Second, associations are encoded by convergence of multiple stimuli onto a common target. For example, association cortex integrates multiple sensory modalities and enables learning about the relationship between them. Finally, learning can be modified by the state of excitability. Fear or arousal, likely through increased levels of monoaminergic neurotransmitters, can more strongly encode the association of a stimulus with the presence of a predator than when an individual senses safety [3].

**Figure 1:**
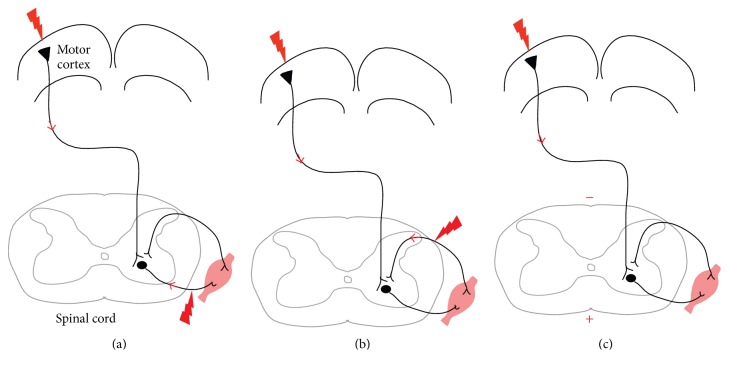
Three paired stimulation models. The corticospinal tract (CST) connects motor cortex directly to the spinal cord. The termination of the CST is largely (>80%) onto interneurons in humans and exclusively so in rodents, but for simplicity it is depicted only as synapsing onto motoneurons. (a) Pre-post synapse model. Repetitive paired stimulation of a presynaptic neuron and its postsynaptic target modifies the strength of the synapse connecting them. The timing of pre-post synaptic neuron firing determines whether the synapse is made stronger or weaker. This is also termed Hebbian or spike-timing dependent plasticity. For corticospinal modulation, this strategy usually pairs motor cortex stimulation with back-propagating peripheral motor nerve stimulation [1, 77–79]. (b) Convergent model. Two (or more) presynaptic neurons converge onto a common postsynaptic target. For corticospinal modulation, this strategy may pair motor cortex stimulation with afferent sensory nerve stimulation [87, 94]. (c) Phasic during tonic model. Adding tonic direct current stimulation concurrently with phasic stimulation at one or more sites can augment corticospinal circuit responses [95, 98, 99]. In this schematic, CST activation of motor cortex is modulated by direct current stimulation of the spinal cord.

Paired electrical stimulation for corticospinal system modulation has evolved to emulate these fundamental learning mechanisms with the goal of enhancing motor function. In this review, we group various paired stimulation paradigms that have been developed for modulation of the corticospinal tract (CST) into three fundamental strategies (Figure 1). First, we discuss Hebbian plasticity in experiments designed to modulate synapses directly between presynaptic corticospinal neurons and postsynaptic spinal motor neurons (Figure 1(a)). The second strategy uses convergent inputs of paired stimuli onto a common postsynaptic target (Figure 1(b)). Sites for this modulation include cortex and spinal cord, usually achieved by pairing corticospinal and afferent stimulation, but at different interstimulus latencies. Finally, we discuss tonic stimulation at one site, for example, the spinal cord via direct current stimulation, to strengthen the effects of phasic stimulation at another site, for example, the motor cortex (Figure 1(c)). In all of these strategies, factors such as relative timing, intensity, and frequency of paired stimulation play crucial roles in determining the direction and duration of circuit modulation.

The review begins with a discussion of the organization of the CST and the different types of single-site modulation. We then review the application of paired stimulation through Hebbian, convergent, and tonic models. Like all models, these three models of paired stimulation are both useful and flawed. They are useful in that they illuminate general principles of paired stimulation interactions on a systems neuroscience level. This enables comparison of different stimulation protocols and emergence of common mechanisms. At this early stage, the models are flawed and incomplete because although a vast body of work has elucidated key synaptic learning mechanisms in vitro, it is difficult to pin down the basic synaptic mechanisms by which paired stimuli interact in the living organism. In addition, paired stimulation protocols may employ more than one overlapping mechanism to achieve modulation. Finally, even pairing aimed at specific circuit nodes likely act at several points in the network. But we believe that a conceptual framework organized into these three models is helpful to build mechanistic understanding and to identify patterns of effective neuromodulation.

## 2. The CST as a Target for Modulation with Paired Stimulation

The CST is a popular target for neuromodulation, in part, because it is so important for human health and function. The corticospinal tract is the direct connection between motor cortex and circuits within the spinal cord and is the principal pathway for skilled voluntary movement, particularly of the hands [4, 5]. Lesion of the CST strongly correlates with motor impairment [6]. In the setting of injury or disease, spared CST connections are largely responsible for motor recovery [7–10]. The CST's roles both in learning and executing motor skills and in providing the substrate for relearning skills after injury indicate that it is a malleable system [11]. This plasticity can occur within the CST itself [12] or as a result of spinal plasticity [13].

The CST is also an attractive target because it is accessible to external electrical stimulation. The motor cortex lies on the convexity of the cerebral hemispheres; the face and arm/hand representations are nearest to the scalp. This enables these regions to be stimulated noninvasively across the scalp, or without disturbing other neural tissues if electrodes are placed above (epidural) or below (subdural) the overlying dura mater. Although spinal motor circuits lie deeper in the body, the spinal cord is still accessible through direct electrical stimulation or indirectly through stimulation of peripheral nerves. In humans, peripheral nerves and nerve roots are the most common targets for modulation of the spinal cord, through either orthodromic activation of afferent fibers or antidromic activation of efferent nerves.

A simplified schematic of the CST is shown in Figure 1. Importantly, the cortical motor system does not exclusively originate from primary motor cortex. Premotor, supplementary motor, and other cortical areas also make large contributions to descending CST pathways, with recurrent connections between motor cortex, sensory cortex, and thalamocortical pathways [14–17]. In the spinal cord, the CST largely terminates onto interneurons of the deep dorsal horn and the intermediate zone [15, 18, 19]. In primates, a small percentage of CST terminations contact motoneurons directly (15–20% in humans); with a few possible exceptions, direct cortex to motoneuron connections is present in rodents only early in development [20, 21]. CST terminations overlap extensively with terminations of large-diameter afferents, which encode joint position, muscle spindle tension, and other sensory modalities critical to skilled movement [22]. The extensive overlap of CST and afferent sensory terminations within the spinal cord provides a substrate for convergent modulation and plasticity of spinal motor circuits using paired stimulation.

While the spinal cord has been regarded in the past as a relatively simple conduit between the brain and periphery, it is now clear that the spinal cord harbors its own complex intrinsic circuitry [23]. Spinal circuits enable skilled movement through the coordination of agonist, antagonist, and stabilizing muscles across body regions [24–26]. A dramatic demonstration of this intricate circuitry is the ability of the lumbar spinal cord to support locomotion in spinalized animals. The intrinsic circuitry mediating these complex acts is termed the locomotor central pattern generator [27]. When given lumbar epidural stimulation, monoaminergic neurotransmitters, and afferent input below the injury, cats [28] and rats [29] with no brain-to-spinal cord connections can stand, walk, and even adapt to obstacles and changes in treadmill direction. Under these conditions, spinal circuits can respond to training by altering synaptic connections and strength, resulting in functional improvements in standing or walking [30, 31].

These results provide information about the use of paired stimulation in three ways. First, intrinsic spinal programs can be modified with experience: like the brain, the spinal cord can learn [32]. This makes the spinal cord an attractive target for modulation. Second, inputs from the brain, sensory afferents, or external electric stimulation do not need to encode the complexity of movement; rather, they can trigger intrinsic spinal cord motor programs to carry out those actions [25, 33]. Third, injury to the brain or spinal cord usually spares a portion of CST and other inputs to the spinal cord [34, 35]. The goal of external stimulation is to enhance connectivity made by these spared neural inputs onto intrinsic spinal circuits.

## 3. Stimulation Modality

To understand the effects of paired stimulation on the CST, we must first understand how single-site stimulation affects the system. Some basic technical concepts regarding individual stimulus modalities are introduced below, as the biophysics of each type of stimulation helps to determine which to use for paired stimulation. Stimulation is largely applied in one of two ways: phasic (short pulses lasting on the order of milliseconds) or tonic (applied at the same intensity over a period of minutes). The amplitude, position, orientation, and polarity of stimulation determine whether pulsed or tonic stimulation produces action potentials in the underlying tissue. In practice, phasic stimuli are more often used to depolarize underlying neurons synchronously with the stimulation; this may be performed repeatedly. On the other hand, tonic stimulation changes tissue excitability, which can alter the firing rate of neurons but without temporal specificity [36].

There is often a trade-off between stimulation focality and invasiveness: direct invasive stimulation on or within a neural target delivers more focal stimulation compared with noninvasive stimulation on the skin. However, even highly localized stimulation, such as that delivered with a sharp electrode into the central nervous system, has effects that spread throughout the interconnected neural circuits [37]. A potential advantage of paired stimulation over unpaired stimulation is that the effects may be constrained by the interaction between the two stimulation sites. This means that diffuse stimulation at one site might gain specificity through interactions at another site. The effects of stimulation intensity are also likely to be complex; like drug therapy, more is not necessarily better. This is true, in part, because more intense stimulation produces less targeted effects. Another potential advantage of paired stimulation is that the synergistic effects of stimulation at separate sites may allow lower stimulation intensity at each site compared to single-site stimulation.

*Magnetic stimulation* uses a transient focal magnetic field to induce a current in the neural tissue underlying the stimulating coil. When placed over the motor cortex, transcranial magnetic stimulation (TMS) can produce CST action potentials. The largest and fastest component of the motor-evoked potential (MEP) travels via the CST [38, 39]. However, polysynaptic pathways involving the reticulospinal, propriospinal, and other tracts contribute as well [40]. TMS offers the advantage of relatively high spatial and temporal specificity. Cortical TMS can be used diagnostically to measure CST function by testing the threshold to provoke motor responses as well as the amplitude, latency, and spatial distribution of those responses. As an intervention, repetitive TMS (rTMS) modulates brain function in rate-dependent fashion. Patterned “theta burst” rTMS (bursts of 3 pulses at 50 Hz, given at a rate of 5 bursts per second) may produce longer-lasting changes in cortical excitability [41, 42]. Excitatory rTMS (repetition rate of 1 Hz or greater) has been applied most extensively in stroke, where it has had a tendency to strengthen TMS-evoked responses and to improve some aspects of arm and leg function [43–45]. Inhibitory TMS (repetition rate of less than 1 Hz) has also been used effectively to dampen activity in the uninjured hemisphere after stroke and thereby reduce interhemispheric inhibition [46].

TMS can also be applied to the spinal cord by holding the stimulating coil over the back of the subject. This approach likely recruits radicular inputs onto spinal cord circuits [47, 48]. For paired stimulation, this approach could be used to recruit afferents in the segment of the spinal cord underlying the stimulating coil, while a TMS coil over motor cortex could be used to stimulate motor cortex and the CST.

*Direct current stimulation (DCS)* uses surface electrodes to deliver continuous low intensity (e.g., 1-2 mA) electric current. A critical difference between DCS and TMS is that DCS delivers* tonic, sub*threshold stimulation rather than directly triggering action potentials. Although only a small fraction of the current crosses the skin, and despite the lack of direct evidence for which circuits DCS activates, data from numerous studies have suggested that DCS modulates underlying neuronal excitability [49–56]. Additionally, DCS has been used over the spinal cord, with possible effects on motor recruitment, pain, and spasticity [57–61]. DCS offers the advantages of lower cost and higher portability than other stimulation techniques. However, several major gaps in mechanistic understanding persist: there is no technique to directly map how the low-energy current is distributed within the body, which neural circuits are activated, or how individual variations in injury characteristics affect DCS circuit activation. Furthermore, the continuous nature of DCS means that it cannot be employed for timing dependent synaptic changes.

*Intraspinal electrical stimulation* through implanted electrodes is used to deliver* phasic* pulses at sub- or suprathreshold intensity directly to the spinal cord. This method is currently limited to animal models due to its highly invasive nature [62].

*Epidural electrical stimulation* delivers* tonic* pulses to the dorsal surface of the spinal cord that are usually subthreshold for activating motor neurons. This stimulation (usually in the range of 15–60 Hz) activates large-diameter sensory afferent fibers that enter the dorsal spinal cord and synapse onto interneuronal and motor circuits [63]. Although subthreshold epidural stimulation alone does not induce any movement, when combined with physical training or monoaminergic drug exposure, SCI animals and human subjects with implanted lumbar epidural stimulators have shown dramatic increases in volitional control of leg muscles below the injury level [29, 64–67]. Whether epidural stimulation directly facilitates increased responsiveness of spinal motor circuits, or whether individual epidural pulses stochastically interact with descending volitional signals to mediate spike-timing dependent synaptic plasticity, remains to be determined. These alternative hypotheses are not mutually exclusive.

*Transcutaneous spinal electrical stimulation* is applied noninvasively, usually at suprathreshold intensities (unlike DCS or epidural stimulation). At the lower range of stimulation intensity, this stimulus modality is thought to activate dorsal afferent fibers, whereas, at higher intensities, transcutaneous stimulation directly activates ventral efferent fibers [68–71]. For example, transcutaneous stimulation over the T11 level at 3 Hz induced coordinated walking movements in uninjured volunteers [47]. Adding simultaneous stimulation at the C5 and L1 levels (at 5 Hz) increased the coordination and range of motion achieved [72]. Delivered at a higher rate (50 Hz) and lower intensity (70% of motor threshold), lumbar transcutaneous stimulation reduced leg spasticity in three subjects with chronic incomplete SCI [73]. The most appealing aspect of this method is its noninvasiveness and portability; using simple adhesive electrodes, transcutaneous spinal stimulation could be given within the context of structured physical rehabilitation exercises.

## 4. Paired Stimulation Strategies

All paired stimulation paradigms share the same objective: to alter connections between specific target circuits. Relative to single-site stimulation, in which activation may spread to other areas connected to the target, paired electrical stimulation may narrow the effect to the site of interaction between multiple stimulation sites. Repetitive paired stimulation at two or more sites is designed to trigger lasting plasticity through synergistic mechanisms. Further considerations include site (brain, spinal cord, and peripheral nerve, each with varying levels of specificity) and whether stimulation is geared toward pre-post synaptic or convergent synaptic summation mechanisms. As demonstrated through decades of research in cellular and slice models, other major variables involved in paired stimulation include timing, intensity, and frequency [74].

Devising paired stimulation paradigms for neuromodulation of the CST involves integrating the systems neuroscience of sensorimotor interactions in the cortex and spinal cord with understanding of the biophysics of the stimulation modality. This is a necessarily iterative process because paired stimulation provides insight into interactions that cannot be achieved otherwise. Instead of a systematic review of the literature, we will highlight selected studies that demonstrate key concepts of using paired stimulation to target specific synaptic connections in animal models and humans. We will discuss progress and describe the main challenges that need to be addressed for paired stimulation to be successfully implemented in human neurological conditions.

*Pre-Post Synaptic Stimulation*. The classic Hebbian approach involves stimuli delivered in synchronous fashion directly to the two neurons connected by the target synapse; coordinated firing of a presynaptic neuron and its postsynaptic target adaptively alters the synapse that connects them [1]. This concept has been advanced experimentally in the hippocampus and other well-understood circuits, where it has been termed spike-timing dependent plasticity (STDP) [2, 74, 75]. We choose to call this approach “pre-post synaptic stimulation” because STDP and Hebbian plasticity have come to mean different things to different people.

The relative delay between pulse arrivals at pre-post synaptic sites dictates whether repetitive paired pulses potentiate or depress the targeted synapse. Work in cellular and slice models has shown that long-term potentiation (LTP) occurs after repetitive pulse arrival at an excitatory presynaptic terminal up to 20 ms prior to pulse arrival at the postsynaptic terminal, whereas long-term depression (LTD) occurs after repetitive pulse arrival at the postsynaptic terminal between 20 and 100 ms prior to the presynaptic terminal [74, 76]. Note that, in vivo, consideration must be given to the latency between stimulation site and synapse arrival. These latencies vary depending on factors such as a subject's height, injury severity or disease status, and effort. Therefore, the interval between two stimulus sites/modalities may need to be individualized based on these factors and the desired site of synaptic interaction. Likewise, the relative intensity of the pre-post synaptic stimuli may affect the polarity and degree of synaptic modulation.

Nishimura and colleagues demonstrated pre-post synaptic stimulation in the CST of healthy primates. To test whether exogenous time-linked spinal stimulation would cause lasting modulation of corticospinal transmission, the investigators used intracortical electrodes to record activity in corticospinal motor neurons during free behavior [77]. Neurons that fired during specific arm movements were used to trigger delivery of intraspinal stimuli to cervical spinal motor neurons controlling the arm muscles that mediate the intended movements. When the latency between endogenous cortical spike and exogenous spinal stimulation was between 12 and 25 ms, corticospinal transmission (as determined by the correlation between cortical motor neuron spike activity and EMG facilitation) increased for at least 24–48 hours* after* stimulation. Conversely, when the investigators varied the timing such that spinal stimulation occurred several ms prior to arrival of the endogenous cortical signal, subsequent corticospinal transmission was depressed. Both of these time windows follow rules established in numerous classic Hebbian experiments [76].

In humans, the pre-post synaptic approach is best exemplified by pairing TMS with motor nerve stimuli such that the pulses arrive synchronously at synapses between corticospinal neurons and motoneurons within the spinal cord (Figure 1(a) and [78–80]). High-intensity electrical stimuli of peripheral nerves innervating arm or hand muscles travel antidromically to motoneurons in the cervical cord. In able-bodied volunteers and subjects with incomplete cervical SCI, a series of 50–90 TMS-peripheral nerve stimulation pairs timed such that TMS pulses arrived at cervical motor neurons 1-2 ms prior to retrograde nerve stimuli led to increased hand muscle motor-evoked potential amplitudes and fine hand dexterity for roughly 30 minutes after stimulation. Reversing the timing (peripheral stimulus arrival at cervical motor synapses 5–15 ms before TMS pulse arrival) resulted in either the opposite or no effect [78, 79]. Encouragingly, application of paired stimulation in the pre-post sequence resulted in transiently increased hand function, not just electrophysiological transmission. In able-bodied subjects, Janet Taylor's group observed increased strength of the targeted biceps muscle [78]. In both able-bodied subjects and those with incomplete cervical SCI, Monica Perez's group observed increased strength and EMG activity in the targeted first dorsal interosseous muscle, as well as increased agility on a skilled pegboard task [79].

Critically, as already described above in primate models, exogenous cortical stimulation could potentially be replaced by using endogenous cortical signals as the presynaptic pairing modality. The intent to move can be detected from intracortical (or less invasive scalp) electrodes and then used to trigger synchronized exogenously delivered spinal or peripheral stimuli. This volitionally driven approach could be used to* amplify* synaptic transmission within incompletely damaged native circuits. This is in distinction from the use of brain-computer interfaces as bypass routes to* replace* function of completely disconnected native circuits. As a large number of brain and spinal injuries spare at least some degree of volitional muscle activation, real-time electromyography (EMG) of the target muscle could serve as a simpler proxy for cortical intent, as demonstrated in rodent models [62]. In humans, an inverted approach has been tested, in which exogenous cortical stimulation is driven rather than replaced by peripheral signals. For example, TMS has been synchronized either with peripheral EMG activity or with timed physical arm movements, with mixed results [81–83].

The pre-post synaptic model represents the most straightforward approach to paired stimulation of the motor system, with timing and other parameters being well-delineated in slice and hippocampal models. However, the mechanistic challenge, especially in the case of volitionally driven human studies, is that it may be difficult if not impossible to precisely determine the circuit identities and synaptic mechanisms that contribute to observed changes in function. In the living organism it remains to be determined whether stimulation can be delivered precisely enough to modulate the targeted synapse without resulting in unintended collateral plasticity.

*Convergent*. In the convergent approach, rather than pairing stimulation between a single presynaptic neuron and postsynaptic neuron, stimuli are delivered to two or more presynaptic neurons that independently synapse onto a common postsynaptic target, resulting in summation of temporally paired inputs (Figure 1(b)). This mechanism was initially described in simplified in vitro and ex vivo preparations from* Aplysia* and neonatal rat spinal cord, where repeated paired activation of separate converging inputs facilitated responses of common target neurons to test stimuli [84–86]. In the living organism, all forms of external stimulation may in fact be at least partially “convergent,” given the difficulty of limiting stimulation precisely to single pre-post synaptic neurons.

In the most highly cited demonstration of paired stimulation in humans, Stefan and colleagues paired median nerve electrical stimulation with TMS over the motor cortex area representing the abductor pollicis brevis muscle, a paradigm dubbed paired afferent stimulation (PAS) [87]. The median nerve was stimulated 25 ms before TMS to allow the median nerve signal to reach the motor cortex, presumably through ascending sensory projections to sensory cortex and then via cortico-cortico connections. A single pair of pulses delivered every 20 seconds for 30 minutes (90 pulses) resulted in increased cortical motor-evoked potential amplitudes at both the abductor pollicis brevis and abductor digiti minimi muscles; augmentation lasted for at least 30 minutes after pairing.

The site of PAS plasticity is likely in the cortex. There was no change in subcortical motor-evoked potential amplitude or in F-wave responses, arguing against a subcortical or spinal locus of plasticity [87]. However, subsequent reports suggest some spinal cord changes in segmental reflexes (paired associative stimulation induces change in presynaptic inhibition of Ia terminals in wrist flexors in humans [88]). The timing dependent sensitivity of PAS was demonstrated by observing no effect when longer ISIs separated the median and TMS pulses and a* decrease* in median nerve-evoked sensory potentials when timing was reversed such that the TMS pulse arrived at somatosensory cortex 10–15 ms prior to the median nerve-evoked potential [89]. These time windows for synaptic potentiation and depression overlap with those seen in Hebbian pre-post synaptic plasticity, demonstrating the universal importance of timing in synaptic plasticity.

Although sometimes characterized as Hebbian, paired associative stimulation is more consistent with the convergent approach. Neither the afferent median nerve electrical impulse nor the cortical magnetic impulse takes direct routes to the target synapse: the afferent peripheral pulse synapses at the brainstem, thalamus, and sensory cortex before traversing intracortical fibers that are input onto pyramidal motor neurons. The TMS pulse also transits through intracortical fibers that converge onto the same pyramidal motor neurons [87, 90]. Thus, these stimuli lead to convergence of two or more presynaptic signals onto a common postsynaptic target—in this case, corticospinal motor neurons in layer V of motor cortex.

Convergence can be targeted to spinal rather than cortical circuits by altering stimulus latencies. For example, in the human, a motor cortical stimulus takes roughly 5–8 ms to reach synapses in the cervical spinal cord and 10–15 ms to reach synapses in the lumbar cord via the CST [79, 91–93]. Synchronized stimuli to afferent sensory inputs converge with descending corticospinal signals onto postsynaptic spinal motor neurons, modulating motor neuron responses depending on relative timing, intensity, and pattern. For example, a paradigm dubbed spinal associative stimulation (SAS) combines subthreshold cortical TMS pulses timed to arrive at soleus motor neurons roughly 5 ms prior to arrival of suprathreshold tibial nerve afferent pulses [94]. Pairing the pulses every 10 seconds for 15 minutes (90 pulse pairs) significantly increased tibial nerve H-reflex amplitude and sensitivity during and immediately after the stimulation period [94]. Whereas this paradigm increased H-reflex amplitude, F-waves were not measured, so the mechanism of increased spinal reflexes is unknown. Furthermore, postintervention TMS motor-evoked potentials were not reported, leaving the question open of whether corticospinal circuits were modulated. Another study targeting SAS toward cervical levels using suprathreshold TMS in combination with median nerve stimulation saw no change in the primary outcomes of TMS-evoked potentials and grip strength. The authors speculated that in this case the paired stimuli may have reached separate rather than common postsynaptic targets.

Convergent paired stimulation has several advantages as well as possible disadvantages compared with pre-post synaptic stimulation. The convergent approach has the advantage that spinal targets are more easily accessed via sensory afferent input than through antidromic motor stimulation, especially because the former can be delivered at lower (and more tolerable) stimulation intensities. In addition, sensory circuits are more easily accessible to surface (e.g., epidural) stimulation of the spinal cord. In addition, lower-intensity sensory stimulation may be easier to integrate with simultaneous physical rehabilitation exercises, providing an opportunity to supplement or supplant exogenous cortical stimulation with endogenous volitional motor signals. On the other hand, the convergent approach may have the disadvantages of more off-target effects and increased complexity by adding other synapses and circuits into the classic two-neuron pre-post synaptic picture.

*Tonic during Phasic*. Both pre-post synaptic and convergent plasticity rely on proper synchronization of paired stimulation on the order of milliseconds. In contrast, tonic stimulation is applied continuously over the course of minutes. Direct current stimulation (DCS) represents the most widely used form of tonic stimulation. For DCS, the positioning and polarity of stimulation, rather than timing, are critical to its effects. We will discuss tonic stimulation of the CST employing transspinal DCS (tsDCS; Figure 1(c)). The induced electric field of tsDCS alters the properties of the spinal cord, modulating responses to brain stimulation and spinal reflexes. Whether the cathode is placed dorsally and the anode ventrally (as shown in Figure 1(c)) or the polarity is opposite (cathode ventral and anode dorsal) has a major impact on the effects.

Both rodent and human experiments demonstrate effects of tsDCS on motor responses evoked by CST stimulation. In rodents, stimulating electrodes are placed subcutaneously to prevent the animal from removing the electrode. In humans, the electrodes are placed on the skin. The sites of stimulation include the neck, torso, and lower back. Mathematical modeling of current flow within the body suggests that the site of stimulation is critical to which peripheral nerves or spinal cord segments are affected by tsDCS [95]. Electrode size and stimulation amplitude, which together determine the current density, are other determinants of the effects of tonic stimulation [96].

A robust and reproducible finding across studies is that tsDCS causes greater augmentation of CST responses when the cathode is placed on the dorsal aspect and the anode ventrally (referred to as cathodal tsDCS). Tonic tsDCS has effects both during the stimulation period and for a period of minutes afterwards. The influence of polarity is particularly strong on the after effects, with cathodal tsDCS causing lasting augmentation of CST motor responses [97]. These effects are mediated by alterations in spinal cord synapses and axonal connections. Thus, cathodal tsDCS can augment CST motor responses when applied as single-site modulation.

The crucial question for paired stimulation is whether tonic tsDCS modulates the effects of concurrent phasic CST neuromodulation. Experiments in the John Martin Laboratory demonstrate that cathodal tsDCS strongly enhances the neuromodulation caused by repetitive motor cortex stimulation in rats. The brain stimulation paradigm used in these studies is intermittent theta burst stimulation, a paradigm involving “bursts” of three stimuli applied at 50 ms intervals with electrodes implanted over motor cortex. As a single modality, theta burst stimulation causes lasting augmentation of CST responses both in rodents and in humans when applied via TMS [41, 98]. When paired with cathodal tsDCS in rats, the slope of theta burst augmentation increased. That is, tonic stimulation of the spinal cord caused larger increases in CST responses than theta burst motor cortex stimulation alone [98]. These effects lasted at least 30 minutes after paired stimulation was applied.

Importantly, pairing tonic with phasic stimulation improves CST* function* and motor skill in rodents with injury. Song et al. employed a cut lesion of the CST emanating from one hemisphere and paired intermittent theta burst stimulation of the spared CST with cathodal tsDCS over the cervical spinal cord beginning the day after injury, similar to a brain stimulation only protocol that was effective [19]. Paired motor cortex intermittent theta burst stimulation and cervical tsDCS were administered for 27 minutes a day for 10 days. This caused a decrease in the number of foot faults while walking across a horizontal ladder; improvement relative to sham tsDCS was sustained throughout the testing period of 31 days. In addition, the threshold of motor cortex stimulation to produce a motor response went* down* by more than 25% (indicating stronger CST responses) whereas the threshold for provoking responses in rats with sham tsDCS went* up* more than 50%. Finally, the protocol produced large-scale sprouting of spared CST axon endings in the gray matter of the cervical spinal cord; the cumulative axon length on the animals' impaired side was more than 5 times that of rats with sham tsDCS. Thus, this tonic during phasic protocol produced robust behavioral improvement that was accompanied by strengthening of CST physiology and function and abundant sprouting into largely denervated regions of the spinal cord.

Since tsDCS can enhance cortical neuromodulation, it may also increase the gain of other neuromodulation strategies. This includes corticospinal neuromodulation based on pre-post synaptic and convergent input. Experiments in Ahmed's laboratory have tested this hypothesis in the lumbar spinal cord of the mouse. One convergent input paradigm paired sciatic nerve stimulation with motor cortex stimulation (similar to PAS, but in the hind limb). When the sciatic nerve was repetitively stimulated up to 120 ms before brain stimulation, subsequent unpaired cortical test pulses were enhanced, demonstrating the lasting augmenting effect of pairing [99]. This convergence paradigm was then performed under tonic cathodal tsDCS, with markedly stronger augmentation of subsequent cortical test pulses. The effect of combining the convergence paradigm and tonic stimulation was larger than predicted by the individual effects, suggesting the synergistic potential of combining tonic stimulation with phasic paired stimulation strategies.

This protocol produced improvements in skilled locomotion in mice with spinal cord hemisection. Stimulation at cortex, sciatic nerve, and tsDCS was delivered beginning 13 days after hemisection at the caudal end of the thoracic spinal cord. Skilled locomotion was assessed using the horizontal ladder, similar to the Song et al. study. This protocol produced large-scale recovery of skilled locomotion; errors in hind limb stepping were reduced 77% in rats with stimulation compared to injury-only animals. Two groups of control mice (tsDCS only and paired motor cortex and sciatic nerve stimulation only) were reported to have improved less, although the data from these mice were not shown [99]. Together, these results suggest that adding tonic spinal cord stimulation increases the physiological and behavioral efficacy of motor cortex and peripheral nerve stimulation.

Clearly, this stimulation paradigm does not conform to the precisely time-locked Hebbian model of paired exogenous stimulation. Whether tonic stimulation itself prepares spinal motor circuits to become more responsive, or whether individual pulses stochastically interact with descending volitional signals at the correct synaptic latency, remains to be determined. Again, these scenarios are not mutually exclusive.

## 5. Gaps and Hurdles

Paired stimulation of the corticospinal system holds unique promise not only for gaining insight into systems-level organization of intact and injured motor control circuitry, but for potential application toward humans with neurological injury and disease. It also offers the possibility of modulating the CST in a circuit-specific manner, in which the effects of pairing are largely restricted to the site of interaction between two stimuli. The promise of paired stimulation is that its potential selectivity may boost efficacy and limit off-target effects, similarly to molecular medicines that specifically bind strongly to their target and limit side effects.

In order to clear the many hurdles impeding application of paired stimulation to humans for therapy, work is ongoing to address these critical questions.

*Is Paired Stimulation Actually “Better” Than Unpaired Stimulation*? This question has only been partially addressed by some of the paired stimulation studies highlighted in this review. These studies compared the effects of varying interstimulus intervals on acute outcomes, mostly related to electrophysiological rather than clinical function. As the paired stimulation field matures, more studies need to compare the effects of paired versus unpaired stimulation across multiple sessions, on meaningful clinical outcomes, in humans with relevant neurological conditions. It is critical to directly compare paired stimulation to unpaired (or sham) stimulation. In particular, for protocols that rely on precise timing of pairing, the most appropriate control will use paired stimulation, but at intervals that are ineffective at producing short-term physiological or behavioral changes.

*How Does Stimulation Duration Influence Effect Duration*? Most stimulation sessions last on the order of minutes. Effects have been measured over periods ranging from immediately after a single session to hours, days, or weeks after completion of multiple sessions. Some of these paradigms and schedules have been based on results of in vitro experiments of synaptic plasticity. Other schedules have been chosen to maximize convenience in human subjects. In many cases, stimulation schedules were chosen empirically and then reproduced in subsequent studies while varying other factors. Is this an optimal approach? The entire field of neurostimulation desperately needs a more systematic approach to defining optimal stimulation schedules. How long should an individual session be? How many sessions should be applied? What are the best intersession intervals [100, 101]? To address these questions, an important assumption first needs to be validated: are short-term physiological effects predictive of long-term behavioral effects? If so, then baseline experiments can focus on short-term physiological effects and then subsequent experiments would aim to optimize longer-term physiological and behavioral effects. These studies would systematically alter the duration and frequency of stimulation in order to maximize the lasting effects. Optimization of these protocols should be a goal for the field.

*How Do Relative Frequency and Intensity of Paired Stimuli Affect Outcome*? Extensive literature documents the effects of interstimulus interval, frequency, and intensity when using single-site stimulation such as TMS. However, there is no clear consensus or formula that dictates which frequency or pattern to use for specific paired scenarios, or how to titrate relative intensity between two stimulation sites. To date, more attention has been directed toward the relative timing of paired stimuli arrival at target synapses. More effort needs to be devoted to optimizing paired pulse frequency and intensity in order to improve paired stimulation efficacy.

*How Can Target and Off-Target Effects of Paired Stimulation be Monitored in Real Time*? Stimulation of one node of a highly interconnected network makes it impossible to confine the effects exclusively to a target pathway. A more realistic goal is to maximize on-target relative to off-target circuit activation. To do this, we need to better understand the networks that are activated by paired stimuli. Ideally, this would involve visualization (or detection) of synaptic events in real time. For analysis of affected circuits, animal models offer advantages of invasive electrophysiology and imaging of neural activity within tracts and at synapses. This approach is likely to yield insights into the systems-level mechanisms of paired stimulation.

While animal studies can provide fundamental insight, the systems mechanisms of paired stimulation must also be studied in humans. In part, mechanistic studies are critical because of the myriad differences between humans and laboratory animals in the scale and organization of neural circuits. In addition, the stimulation protocols used in each species differ significantly. Modeling of current flow within tissues and mathematical predictions of circuit effects may prove helpful in translating animal studies to human studies [102–106]. But direct mechanistic studies of local and network effects of paired stimulation in humans are critical. This may involve use of established physiology techniques along with functional imaging of the human nervous system. In this way, mechanistic understanding and functional effects of paired stimulation may be translated from animal models into effective therapy for people with neurological impairments.
